# Abnormal oxygen homeostasis in the nucleus tractus solitarii of the spontaneously hypertensive rat

**DOI:** 10.1113/EP086023

**Published:** 2017-03-05

**Authors:** Patrick S. Hosford, Julian Millar, Andrew G. Ramage, Nephtali Marina

**Affiliations:** ^1^ Center for Cardiovascular and Metabolic Neuroscience, Department of Neuroscience, Physiology and PharmacologyUniversity College LondonLondonUK; ^2^ Barts and the London School of Medicine and DentistryLondonUK; ^3^ Clinical Pharmacology and Experimental Therapeutics, Division of MedicineUniversity College LondonLondonUK

**Keywords:** arterial hypertension, brainstem, neurovascular coupling

## Abstract

**New Findings:**

- **What is the central question of this study?**
  Arterial hypertension is associated with impaired neurovascular coupling in the somatosensory cortex. Abnormalities in activity‐dependent oxygen consumption in brainstem regions involved in the control of cardiovascular reflexes have not been explored previously.
- **What is the main finding and its importance?**
  Using fast‐cyclic voltammetry, we found that changes in local tissue $P_{O_{2}}$ in the nucleus tractus solitarii induced by electrical stimulation of the vagus nerve are significantly impaired in spontaneously hypertensive rats. This is consistent with previous observations showing that brainstem hypoxia plays an important role in the pathogenesis of arterial hypertension.

The effects of arterial hypertension on cerebral blood flow remain poorly understood. Haemodynamic responses within the somatosensory cortex have been shown to be impaired in the spontaneously hypertensive rat (SHR) model. However, it is unknown whether arterial hypertension affects oxygen homeostasis in vital brainstem areas that control cardiovascular reflexes. In this study, we assessed vagus nerve stimulation‐induced changes in local tissue $P_{O_{2}}$ ($P_{tO_{2}}$) in the caudal nucleus tractus solitarii (cNTS) of SHRs and normotensive Wistar rats. Measurements of $P_{tO_{2}}$ were performed using a novel application of fast‐cyclic voltammetry, which allows higher temporal resolution of O_2_ changes than traditional optical fluorescence techniques. Electrical stimulation of the central cut end of the vagus nerve (ESVN) caused profound reductions in arterial blood pressure along with biphasic changes in $P_{tO_{2}}$ in the cNTS, characterized by a rapid decrease in $P_{tO_{2}}$ (‘initial dip’) followed by a post‐stimulus overshoot above baseline. The initial dip was found to be significantly smaller in SHRs compared with normotensive Wistar rats even after ganglionic blockade. The post‐ESVN overshoot was similar in both groups but was reduced in Wistar rats after ganglionic blockade. In conclusion, neural activity‐dependent changes in tissue oxygen in brainstem cardiovascular autonomic centres are significantly impaired in animals with arterial hypertension.

## Introduction

The central nervous system is a major target for the deleterious effects of arterial hypertension. Profound cerebrovascular changes in the brain of hypertensive individuals include vascular remodelling, reduced cerebral autoregulation, white matter lesions, cerebral microbleeds and lacunar infarcts, which are all important risk factors for the development of cognitive impairment, dementia and stroke. In the spontaneously hypertensive rat (SHR) model, magnetic resonance angiography studies have recently shown that the diameter of the lumen in main cerebral vessels becomes progressively smaller, often leading to stenosis (Li *et al*. 2015). As a result, vascular abnormalities created by hypertension may cause a reduction or impairment of activity‐dependent vascular responses (Jennings *et al*. 2005; Calcinaghi *et al*. 2013; Iddings *et al*. 2015). However, the impact of arterial hypertension on haemodynamic responses to neuronal stimulation in the brainstem, a region crucial for cardiovascular homeostasis, remains unknown.

The nucleus tractus solitarii (NTS) is the primary brainstem site of integration of cardiorespiratory reflexes. Mechanosensory nerve endings located in the aortic arch and carotid sinus detect pulsatile flow‐related information, which is transmitted to the NTS via vagal and glossopharyngeal nerves. Increased afferent baroreceptor activity induced by increases in arterial blood pressure (ABP) results in reflex activation of parasympathetic cardiovagal activity and concomitant reduction of sympathetic outflow (with ABP reductions eliciting the opposite response). Electrolytic lesions of cardiovascular control areas in the NTS produce dramatic increases in ABP, leading to sustained arterial hypertension, suggesting that damage to the NTS may play an important role in the development of arterial hypertension (Doba & Reis, 1973; Nathan & Reis, 1977; Snyder *et al*. 1978). We have recently shown that in anaesthetized SHRs, resting local tissue $P_{O_{2}}$ ($P_{tO_{2}}$) in the brainstem is significantly lower compared with normotensive Wistar rats, despite normal values of arterial $P_{O_{2}}$ (Marina *et al*. 2015). However, it is unknown whether $P_{tO_{2}}$ changes that follow increased neuronal input to the brainstem are also abnormal in the SHR. Thus, the purpose of this study was to compare activity‐dependent $P_{tO_{2}}$ changes in the NTS of SHRs and normotensive Wistar rats.

## Methods

### Ethical approval

All animal experiments were performed in accordance with the European Commission Directive 86/609/EEC (European Convention for the Protection of Vertebrate Animals used for Experimental and Other Scientific Purposes) and the UK Home Office (Scientific Procedures) Act (1986) and ARRIVE (Animal Research: Reporting of *In Vivo* Experiments) guidelines with project approval from the University College London Institutional Animal Care and Use Committee. The experimental work was carried out by investigators who understand the ethical principles under which the journal operates, and the work presented herein complies with the journal's ethics checklist. Animals were sourced from Charles River UK and housed at University College London's central biological services facility on a 12 h–12 h light–dark cycle. Animals had access to standard laboratory chow and water *ad libitum*. On completion of the experiment, animals were humanely killed with an overdose an anaesthetic (pentobarbital 60 mg kg^−1^, i.v.; Animalcare, York, UK).

### Experimental procedures

Experiments were performed on seven adult SHRs (15 weeks of age) and five age‐matched Wistar rats. Anaesthesia was induced with isoflurane (2.5% in inspired oxygen; Baxter, Northampton, UK) followed by α‐chloralose (75 mg kg^−1^, i.v. initial dose followed by supplementary doses of 10–20 mg kg^−1^, i.v. as required; Sigma Aldrich, Irvine, UK). The depth of anaesthesia was assessed by the stability of heart rate and ABP. The right femoral artery was cannulated for ABP monitoring and for analysis of pH and blood gases. Blood pressure was measured using a pressure transducer (Neurolog NL108T2; Digitimer; Welwyn Garden City, UK), and heart rate was derived electronically from the ABP signal. The femoral vein was cannulated for drug administration. The trachea was cannulated and the animals were artificially ventilated (rate of 60 strokes min^−1^; stroke volume of the pump was set at 8 ml kg^−1^) with oxygen‐enriched room air using a positive pressure ventilator (Harvard Rodent Ventilator 683; Harvard Apparatus, Cambridge, UK). A neuromuscular blocker (α‐bungarotoxin, 140 μg kg^−1^, i.v.; Life Technologies, Paisley, UK) was infused, and the depth of anaesthesia was assessed by monitoring the stability of ABP and heart rate and the lack of cardiovascular responses to pinching of the paw. Additional anaesthetic was administered when required and arterial blood samples were collected at regular intervals in heparinized capillary tubes and analysed using a pH/blood gas analyser (Siemens Rapidlab^®^ 248; Siemens Healthcare, Sudbury, UK). Arterial $P_{O_{2}}$ was maintained within the physiological range by adjusting the rate and/or stroke volume of the ventilator. It is worth noting that arterial blood pH and $P_{CO_{2}}$ were slightly low in both groups (Table 1), suggesting that rats were experiencing a mild metabolic acidosis, which may have had an impact on resting chemoreceptor drive and sympathetic activity. However, it is unlikely that our conclusions were affected because all SHRs and Wistar rats were in similar physiological conditions.

**Table 1:** Haemodynamic and blood gas values in Wistar and spontaneously hypertensive rats under anaesthesia

Parameter	Wistar rats (*n* = 5)	Spontaneously hypertensive rats (*n* = 7)	*P* Value
SABP (mmHg)	140 ± 4	190 ± 6	0.0043
DABP (mmHg)	83 ± 4	109 ± 5	0.0087
MABP (mmHg)	102 ± 3	136 ± 5	0.0043
Heart rate (beats min^−1^)	372 ± 25	351 ± 11	0.05
pH	7.3 ± 0.02	7.3 ± 0.004	0.16
$P_{CO_{2}}$ (mmHg)	36.1 ± 3.3	34.3 ± 1.3	0.77
$P_{O_{2}}$ (mmHg)	108.8 ± 5	104.6 ± 2	0.77

Data are presented as means ± SD. Statistical comparisons were made with the Mann–Whitney *U* test. Abbreviations: DABP, diastolic arterial blood pressure; MABP, mean arterial blood pressure; and SABP, systolic arterial blood pressure.

Body temperature was monitored via a rectal probe and maintained at 37–38°C with a Homeothermic Blanket Control Unit (Harvard Apparatus).

The rats were placed in a stereotaxic frame and the left cervical vagus nerve was accessed via a dorsolateral incision at the lower neck. The nerve was exposed, separated from the sympathetic trunk and placed on bipolar silver wire electrodes for electrical stimulation (800 μA, 1 ms, 1–5 Hz) using a constant‐current isolated stimulator (Digitimer DS3) triggered by the 1401+ (Cambridge Electronic Design, Cambridge, UK). Distal to this stimulating site, the vagus was crushed and tied. The exposed length of nerve was embedded in dental impression material (Super‐Dent light body dental polyvinylsiloxane; Carlisle Laboratories; Carlisle, UK).

#### Voltammetry measurements in the cNTS

**Figure 1:**
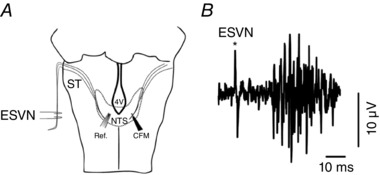
Schematic diagram of experimental set‐up *A*, carbon fibre microelectrodes (CFM) and reference electrodes (Ref.) were inserted in either side of the caudal nucleus of the solitary tract (cNTS). ST, Solitary Tract; 4V, 4th Ventricle. *B*, the location of the electrode in the cNTS was verified by recording evoked potential (multispike) responses from NTS neurons in response to electrical stimulation of the vagus nerve (ESVN).

The dorsal surface of the caudal brainstem was exposed by removing the muscles from the posterior wall of the neck and their insertions from the occipital bone. The dura mater overlying the brainstem was incised and carefully retracted. Carbon fibre microelectrodes were constructed as described before (Millar & Pelling, 2001; Hosford *et al*. 2015). The microelectrode was inserted into the cNTS, the primary relay area of cardiorespiratory vagal afferents, using co‐ordinates taken from the Paxinos & Watson (1998) rat stereotaxic brain atlas (from calamus scriptorius: 0.5 mm lateral, 0.2–0.5 mm rostral and 0.5 mm ventral; Fig. 1 *A*). A Millar voltammeter (Paul Summers Instruments, West Molesey, UK) was used in a three‐electrode configuration (active, Ag–AgCl reference and auxiliary ground). The $P_{tO_{2}}$ was measured by a modified version of differential scan fast‐cyclic voltammetry (Millar & Williams, 1990; Hosford *et al*. 2015).

#### Oxygen detection by voltammetry

A voltage ramp was applied to the carbon fibre microelectrode tip from 0 to −1 V at 200 V s^−1^, four times every second. Molecular oxygen at the surface of the carbon fibre is reduced to water. Electrons for this reaction are supplied by the microelectrode, which cause an increase in cathodic current within a specific voltage range. The current change is proportional to the concentration of oxygen in the parenchyma and can be calibrated to give a semi‐quantitative measure of the analyte.

Unlike the traditional optical fluorescence sensors used in our previous study (Marina *et al*. 2015), voltammetry has significant advantages. As the sensing element is only 7–8 μm in diameter (the tip of a carbon fibre electrode), it can be inserted into the brain tissue using a micromanipulator to a precisely controlled depth with minimal trauma. Thus, oxygen measurements can be made from a precise sterotaxically specified location. The carbon fibre electrode can also be used for extracellular spike recording. Thus, before taking an oxygen measurement the location of the electrode in NTS can be verified by recording evoked potential (multispike) responses from NTS neurons in response to electrical stimulation of the vagus nerve (ESVN; Fig. 1 *B*). These evoked potentials occurred only if the electrode tip was positioned correctly in the NTS. Finally, the sampling rate of four scans per second allowed rapid detection and high temporal resolution of changes in $P_{tO_{2}}$.

Voltammetric scans were triggered continuously and ESVN commenced after a stabilization period of 15 min. Scan output currents were digitized and stored on a PC using a 1401+ and Spike2 software (Cambridge Electronic Design, Cambridge) for analysis offline. The $P_{tO_{2}}$ changes induced by ESVN were taken relative to a 1 s baseline data set preceding application of the stimulus. Electrodes were calibrated before and after the experiment in room air and N_2_‐saturated saline solution at room temperature. Faradaic current corresponding to oxygen content was isolated from background changes.

**Figure 2:**
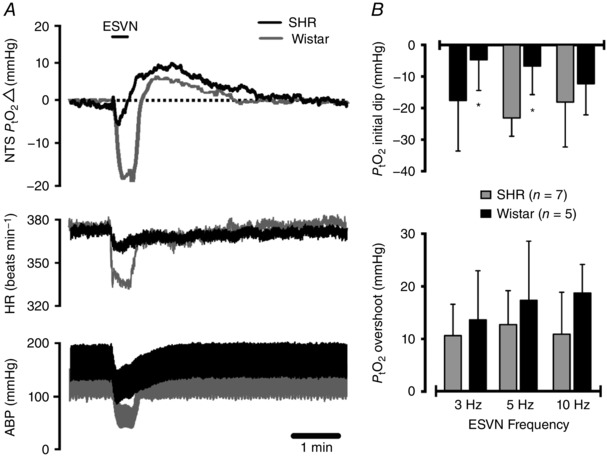
Measurements of tissue partial pressure of oxygen ($P_{tO_{2}}$) in the caudal NTS of spontaneously hypertensive rats (SHRs) and Wistar rats with intact ganglionic transmission *A*, representative experimental trace showing the effect of electrical stimulation of the cut central end of the left vagus nerve (ESVN) on baseline arterial blood pressure (ABP), heart rate (HR) and $P_{tO_{2}}$. Functional activation of the NTS by ESVN caused a profound decrease in arterial blood pressure and heart rate as well as a biphasic change in $P_{tO_{2}}$ characterized by an initial dip followed by an overshoot above baseline. *B*, group data showing the effect of ESVN at different stimulation frequencies. Data are presented as means ± SD. These changes were compared with control values using a two‐way ANOVA and the means compared with Fisher's LSD test.

Electrical stimulation of the vagus nerve was applied at intervals of 10 min at 3, 5 and 10 Hz (frequencies were randomized). In naive animals, ESVN trials were accompanied by profound reductions in ABP and heart rate (Fig. 2 *A*). To examine whether different baseline ABP levels determine the amplitude of $P_{tO_{2}}$ responses between strains and in order to eliminate confounding effects of ABP swings induced by ESVN, ABP levels were clamped in both groups to ∼100 mmHg by administration of the ganglion blocker chlorisondamine (1 mg kg^−1^, i.v.; Sigma‐Aldrich) followed by an infusion of phenylephrine (∼20 μg min^−1^, i.v.; Sigma‐Aldrich).

#### Detection of evoked extracelluar potentials in the cNTS

To determine whether decreased neuronal inputs to the cNTS in the SHRs were responsible for the smaller initial $P_{tO_{2}}$ responses and reduced heart rate changes induced by ESVN, evoked extracellular potentials were simultaneously recorded using the same carbon fibre microelectrode. Total spike activity was integrated in a 20 ms window with a 15 ms delay after each pulse of vagal stimulation and is presented as a function of amplitude over time (in millivolts per second).

### Data analysis and statistics

Stimulus frequency trials were randomized within each animal, and each animal was considered as one repeat. Data were analysed by an experimenter who was blinded to the condition of the animals. Statistical analysis was performed using GraphPad Prism version 6 for Mac OS X (GraphPad Software, La Jolla, CA, USA; www.graphpad.com). The $P_{tO_{2}}$ data are expressed in millimetres of mercury as the mean ± SD and compared using two‐way ANOVA with Fisher's LSD *post hoc* test. Statistical significance was set at the level of *P* < 0.05.

## Results

### Voltammetric measurements of $P_{tO_{2}}$ in the caudal cNTS

Resting blood pressure levels in anaesthetized SHRs were significantly higher than those of Wistar rats (see Table 1). Electrical stimulation of the vagus nerve caused biphasic $P_{tO_{2}}$ changes in the cNTS characterized by a profound decrease (‘initial dip’) in $P_{tO_{2}}$ followed by a post‐stimulus overshoot with respect to baseline (Fig. 2 *A*; *n* = 7 per group). The initial decrease in $P_{tO_{2}}$  was found to be significantly smaller in the SHRs compared with Wistar rats when stimulated at 3 Hz (−5 ± 10 mmHg in SHRs *versus* −18 ± 16 mmHg in Wistar rats, *P* = 0.036) and 5 Hz (−7 ± 9 mmHg in SHRs *versus* −23 ± 6 mmHg in Wistar rats, *P* = 0.019; Fig. 2 *A* and *B*). There was no significant difference in the post‐stimulus $P_{tO_{2}}$ overshoot when stimulated at 3 Hz (14 ± 9 mmHg in SHRs *versus* 11 ± 6 mmHg in Wistar rats, *P* = 0.48), 5 Hz (17 ± 11 mmHg in SHRs *versus* 13 ± 6 mmHg in Wistar rats, *P* = 0.34) or 10 Hz (19 ± 5 mmHg in SHRs *versus* 11 ± 8 mmHg in Wistar rats, *P* = 0.08; Fig. 2 *B*). The effects of ESVN were accompanied by a profound decrease in ABP and heart rate (Fig. 2 *A*). Blood pressure changes were not significantly different between strains when stimulated at 3 Hz (−32 ± 10 mmHg in SHRs *versus* −33 ± 23 mmHg in Wistar rats, *P* = 0.95), at 5 Hz (−41 ± 11 mmHg in SHRs *versus* −41 ± 18 mmHg in Wistar rats, *P* = 0.99) or at 10 Hz (−42 ± 15 mmHg in SHRs *versus* −41 ± 19 mmHg in Wistar rats, *P* = 0.93; Fig. 1 *B*). However, changes in heart rate in response to ESVN were found to be smaller in the SHRs when stimulated at 5 Hz (−20 ± 6 beats min^−1^ in SHRs *versus* −50 ± 31 beats min^−1^ in Wistar rats) and at 10 Hz (−16 ± 10 beats min^−1^ in SHRs *versus* −42 ± 27 beats min^−1^ in Wistar rats), but not at 3 Hz (−17 ± 7 beats min^−1^ in SHRs *versus* −38 ± 30 beats min^−1^ in Wistar rats).

**Figure 3:**
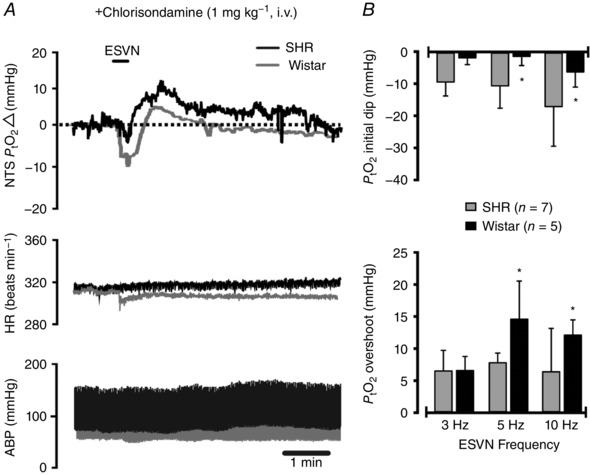
Measurements of $P_{tO_{2}}$ in the caudal NTS of SHRs and Wistar rats under ganglionic blockade Animals received a dose of chlorisondamine (1 mg kg^−1^ i.v.), and mean arterial blood pressure (ABP) levels were normalized to a set value of ∼100 mmHg with an infusion of phenylephrine. *A*, representative experimental trace showing the effect of electrical stimulation of the cut central end of the left vagus nerve (ESVN) on tissue $P_{O_{2}}$ ($P_{tO_{2}}$). The ESVN caused a biphasic change in $P_{tO_{2}}$ in the NTS characterized by an initial dip followed by an overshoot above baseline. Note that during ganglionic blockade, ESVN no longer causes fluctuations in ABP and heart rate (HR). *B*, group data showing the effect of ESVN at different stimulation frequencies. Data are presented as means ± SD. These changes were compared with control values using a two‐way ANOVA and the means compared with Fisher's LSD test.

To explore the possibility that different neurovascular coupling responses in the cNTS might be attributable to increased resting ABP levels in the SHRs and to eliminate confounding effects of ESVN‐induced hypotension on $P_{tO_{2}}$ responses in the cNTS, ABP levels were clamped to 100 mmHg after administration of the ganglion blocker chlorisondamine followed by an infusion of phenylephrine. As a result, resting mean ABP levels were similar in both groups, and ESVN no longer caused rapid changes in ABP or heart rate (Fig. 3 *A*). In these conditions, differences in the amplitude of initial $P_{tO_{2}}$ responses between SHRs and Wistar rats were preserved after ganglionic blockade (−1 ± 3 mmHg in SHRs *versus* −11 ± 7 mmHg in Wistar rats when stimulated at 5 Hz, *P* = 0.048; −6 ± 5 mmHg in SHRs *versus* −17 ± 12 mmHg in Wistar rats when stimulated at 10 Hz, *P* = 0.007), but not at 3 Hz (−1.5 ± 2 mmHg in SHRs *versus* −9.1 ± 4 mmHg in Wistar rats, *P* = 0.07; Fig. 3 *A* and *B*). In contrast, after ganglionic blockade the post‐stimulus $P_{tO_{2}}$ overshoot became significantly larger in SHRs when stimulated at 5 Hz (14 ± 6 mmHg in SHRs *versus* 8 ± 2 mmHg in Wistar rats, *P* = 0.036) and 10 Hz (12 ± 2 mmHg in SHRs *versus* 6 ± 7 mmHg in Wistar rats, *P* = 0.035), but not at 3 Hz (6 ± 2 mmHg in SHRs *versus* 6 ± 3 mmHg in Wistar rats, *P* = 0.035; Fig. 3 *A* and *B*).

**Figure 4:**
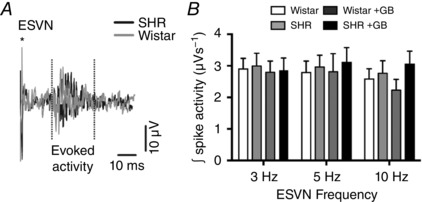
Evoked extracellular potentials in the caudal NTS of SHRs and Wistar rats after electrical stimulation of the vagus nerve (ESVN) *A*, representative trace showing extracellular activity in the caudal NTS following ESVN (^*^denotes stimulus artefact). *B*, group data showing the effect of different stimulation frequencies on integrated spike activity. Data are presented as means ± SD. These changes were compared with control values using a two‐way ANOVA and the means compared with Fisher's LSD test; no significant differences were found. Abbreviation: GB, ganglionic blockade.

Analysis of evoked extracellular potentials in the cNTS showed no significant differences in integrated spike activity between SHRs and Wistar rats at 3, 5 or 10 Hz stimulation frequencies (Fig. 4).

## Discussion

Changes in local brain perfusion in response to increased neuronal activity, a process known as neurovascular coupling, allow the brain to match energy supply with demand. In this study, we used fast‐cyclic voltammetry to measure brainstem $P_{tO_{2}}$ during activation of cardiovascular afferents. An important limitation of voltammetry is its inability to detect baseline $P_{tO_{2}}$ levels. However, this technique allowed us to detect rapid changes in $P_{tO_{2}}$ with substantial spatial and temporal resolution. We compared $P_{tO_{2}}$ responses in the cNTS of untreated hypertensive and normotensive adult rats. Our results provide evidence for an impairment in oxygen homeostasis in brainstem centres involved in cardiovascular control in animals with arterial hypertension.

Upon activation of vagal afferents in both Wistar rats and SHRs, $P_{tO_{2}}$ was found initially to decrease and then overshoot to above prestimulus levels in the post‐stimulus phase. This biphasic response was similar to brain haemodynamic changes reported in studies using different imaging modalities, such as optical imaging of deoxyhaemoglobin (Jones *et al*. 2001) and functional magnetic resonance imaging (fMRI) of blood‐oxygenation‐level‐dependent (BOLD) responses (Menon *et al*. 1995; Kim *et al*. 2000). The initial dip is believed to be the consequence of increased oxygen consumption of local neuronal networks and has been shown to be influenced by basal $P_{tO_{2}}$ levels and metabolic demand of the tissue (Hu & Yacoub, 2012). Stimulation of vagal afferents in SHRs was found to evoke smaller initial $P_{tO_{2}}$ dips in the cNTS as well as smaller heart rate changes than in Wistar rats. There is substantial evidence that both afferent baroreflex sensitivity and baroreflex heart rate responses are significantly impaired in SHRs (Sapru & Wang, 1976; Andresen *et al*. 1978; Head & Adams, 1988; Andresen & Yang, 1989; Minami & Head, 1993; Fazan *et al*. 1999). This suggests that attenuated initial $P_{tO_{2}}$ dips in the cNTS of SHRs during stimulation of vagal inputs may be caused, at least in part, by abnormalities in the baroreflex response or in evoked afferent activity in the NTS (because of decreased synaptic activity). However, analysis of spike activity in the NTS of SHRs and Wistar rats revealed no difference in the evoked volley of action potentials elicited by ESVN even in conditions of ganglionic blockade. This indicates that other factors, such as abnormalities in vertebral blood flow or in tissue oxygen consumption or metabolism might play an important role in the attenuation of initial $P_{tO_{2}}$ responses in SHRs. Indeed, we have previously shown that resting brainstem $P_{tO_{2}}$ in SHRs is significantly lower than in Wistar rats, and brainstem hypoxia in SHRs is exacerbated when ABP levels are lowered to a level similar to that obtained in the present study after ganglionic blockade (Marina *et al*. 2015). As the brainstem of SHRs normally operates at a lower basal $P_{tO_{2}}$, we suggest that an attenuated initial dip in the cNTS would presumably be the result of a smaller O_2_ reserve for utilization when neuronal activity first occurs or general abnormalities with neurovascular coupling in the SHR.

In contrast to the attenuated post‐stimulus initial dip, the cNTS $P_{tO_{2}}$ overshoot following the initial dip was similar in naive SHRs and normotensive Wistar rats. However, this response was found to be reduced in Wistar rats after ABP levels were clamped. In these conditions, ABP in the SHRs was reduced to levels similar to those observed in normotensive Wistar rats, and ABP swings induced by ESVN were largely prevented. This suggests that in normotensive rats, a large component of the post‐stimulus overshoot is dependent on changes in systemic blood flow induced by ESVN, whereas in the SHR, the overshoot is largely preserved and is not affected by underlying ABP levels. The post‐stimulus $P_{tO_{2}}$ overshoot is known to be uncoupled from metabolic demand (Leithner & Royl, 2014) and is determined by increases in local synaptic activity itself and the ability of the neurovascular unit to respond to increases in synaptic transmission (Logothetis *et al*. 2001). However, previous studies have shown that preconstriction of the cerebral vasculature can enhance the BOLD response evoked by somatosensory stimulation (Mulderink *et al*. 2002). Interestingly, it has been shown that SHRs display increased vertebrobasilar artery remodeling and constriction (Cates *et al*. 2011), which may explain, at least in part, why the post‐stimulus overshoot in SHRs is maintained at a similar level before ganglionic blockade, despite operating at a hypoxic baseline level. Alternatively, it may be possible that SHRs have greater capacity to increase post‐stimulus brainstem oxygenation because they are hypoxic at rest or because they have slower O_2_ consumption rates.

In conclusion, our results show that animals with uncontrolled arterial hypertension have impaired oxygen consumption in brainstem areas that control cardiovascular reflexes. These data support our hypothesis that abnormal oxygen homeostasis in the brainstem may play a fundamental role in the pathogenesis of autonomic dysfunction associated with arterial hypertension.

## Additional information

### Competing interests

None declared.

### Author contributions

N.M. and P.S.H. conceived and designed the study. P.S.H. performed the experiments. P.S.H. and J.M. performed data analysis and interpreted data. N.M. drafted the manuscript and acted as corresponding author. A.G.R. supervised development of work, helped in data interpretation and revised the manuscript for important intellectual content. All authors approved the final version of the manuscript and agree to be accountable for all aspects of the work in ensuring that questions related to the accuracy or integrity of any part of the work are appropriately investigated and resolved. All persons designated as authors qualify for authorship, and all those who qualify for authorship are listed.

### Funding

This work was supported by a British Heart Foundation Intermediate Basic Research Science Fellow for Nephtali Marina (grant no. FS/13/5/29927).
